# When students’ words hurt: 12 tips for helping faculty receive and respond constructively to student evaluations of teaching

**DOI:** 10.1080/10872981.2022.2154768

**Published:** 2022-12-06

**Authors:** Susannah Cornes, Dario Torre, Tracy B. Fulton, Sandra Oza, Arianne Teherani, H. Carrie Chen

**Affiliations:** a Clinical Neurology, University of California at San Francisco, San Francisco, California, USA; b Medicine and Director Programs of Assessment, University of Central Florida College of Medicine, Orlando, Florida, USA; c Biochemistry and Biophysics, University of California at San Francisco, San Francisco, California, USA; d Medicine, Albert Einstein College of Medicine, Bronx, New York, USA; e Medicine, Director of Program Evaluation and Education Continuous Quality Improvement, University of California at San Francisco, San Francisco, California, USA; f Assessment and Educational Scholarship, Department of Pediatrics, Georgetown University School of Medicine, Washington, DC, USA

**Keywords:** Student evaluations of teaching, feedback, growth mindset, faculty teaching improvement, faculty development

## Abstract

Student evaluations of curricular experiences and instructors are employed by institutions to obtain feedback and guide improvement. However, to be effective, evaluations must prompt faculty action. Unfortunately, evaluative comments that engender strong reactions may undermine the process by hindering innovation and improvement steps. The literature suggests that faculty interpret evaluation feedback as a judgment not just on their teaching ability but on their personal and professional identity. In this context, critical evaluations, even when constructively worded, can result in disappointment, hurt, and shame. The COVID pandemic has challenged institutions and faculty to repeatedly adapt curricula and educational practices, heightening concerns for faculty burnout. In this context, the risk of ‘words that hurt’ is higher than ever. This article offers guidance for faculty and institutions to support effective responses to critical feedback and ameliorate counterproductive effects of learner evaluations.

## Introduction

Student evaluations of teaching (SETs) are commonly used as part of institutional, course, or clerkship evaluations to guide improvement [1,2]. For SETs to be effective, they must prompt faculty action that involves reflection, professional learning and change [3]. Unfortunately, these actions by faculty may be undermined when SETs engender strong reactions that hinder improvement steps [4,5].

SETs can lead to positive or negative emotions depending both on their content and the faculty experience. SETs can be categorized as critical versus reinforcing according to whether they find fault with or compliment teaching, and as constructive or non-constructive according to whether they facilitate improvement through a non-judgmental, behavior-based framing. Non-constructive, critical SETs fail to elucidate actionable improvements and in rare instances include abusive (0.04%) or unprofessional (0.15%) narrative comments [6]. Critical, constructive SETs are instrumental for improvement, but may still lead to negative faculty emotions, including feelings of disappointment, hurt and shame [4].

Recent events have added stress to faculty’s roles, which may further undermine their productive response to SETs. The COVID pandemic has added anxiety for faculty with clinical roles [7] at the same time that faculty and institutions have been challenged to adapt and readapt their curricula and educational practices to suit a range of remote and hybrid formats [8,9]. Simultaneously, the urgency to develop anti-racist curricula that combat healthcare disparity has meant innovation of content that is emotionally charged. In the setting of increasing concern for faculty burnout, the risk of ‘words that hurt’ is higher than ever.

There is little guidance in medical education for faculty to navigate student feedback that hurts. Such guidance has the potential to support faculty efficacy, promote a greater appreciation for the faculty experience, and enable school leadership to create frameworks that enhance quality improvement. To build feedback literacy [the meta-feedback skills that enable getting the most out of feedback; 10] and to encourage ongoing productive responses to critical feedback, we outline steps to 1) facilitate a shared understanding for the role of learner feedback in the educational endeavor (tips 1–3), 2) foster among the faculty a growth mindset and productive response to words that hurt (steps 4–8), and 3) provide institutions a framework for support and oversight that enable long-term faculty educator improvement and retention (9–12).

## Tips for both faculty and institutions

### Tip 1: appreciate both the complexity of feedback from SETs and its purpose to guide improvement

**Figure 1.**
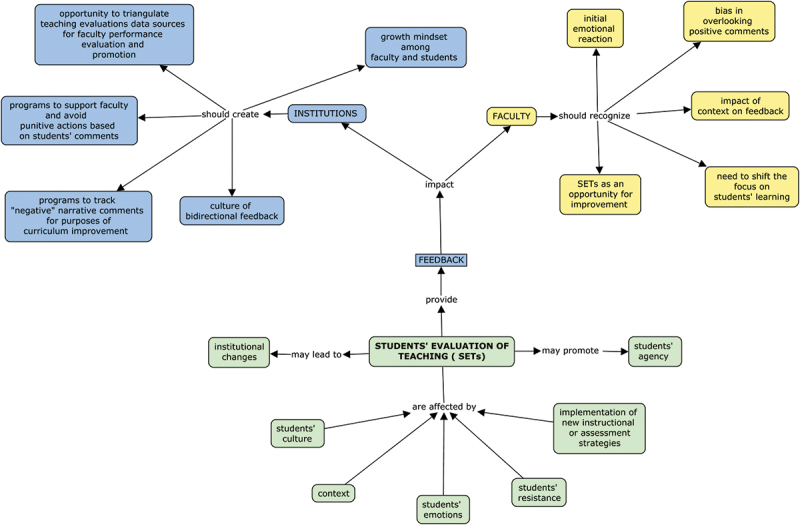
A concept map showing the impact of students’ evaluations of teaching (SETs) on institutions and faculty as well as factors affecting SETs.

Feedback is not just an exchange of information between teacher and learner but an inherently complex educational phenomenon [11] which can be affected by many factors (Figure 1). Both the giving and receiving of feedback are part of a social process influenced by cultural values, personal beliefs, and expectations. At times, SETs may reflect learner dissatisfaction with an unmet expectation that is not related to the specific educational activity. Faculty reconciliation and assimilation of critical feedback with their own individual views is influenced by their professional culture, emotions, reflection, and personal self-efficacy [3,12]. Faculty and institutions should examine these factors as they apply within their working environment and reflect on their interconnectedness before developing a plan for learning and practice change [13].

Despite the complexity of feedback generally, all stakeholders – learners, faculty, and institutions – should appreciate that feedback in the form of SETs is critical informants of continuous quality improvement. Given learners’ proximity to and involvement in teaching events, learners are ideal partners for faculty to identify points of correction, missing or redundant topics, or issues that may hinder learning. In the vein of participatory evaluation approaches, which involve the stakeholders of a program in the evaluation process, evaluation instruments should be developed with the intent to partner with learners and with the goal of enabling improvements rooted in the educational mission [see tip 9; 2, 14, 15].

Feedback should not be a judgment of the faculty’s professional identity, personal qualities or worth [16]. Faculty should engage in perspective-taking [17] and appreciate the distinction between their intent and the impact on learners. For example, if a teacher implements a flipped classroom approach with the intent to promote self-directed learning, but learners perceive it as a lack of guidance and direction, the result is a misalignment between the teacher’s intent and the expected impact. Imagining the learner's perspective helps faculty and institutions model the growth mindset we ask of our learners and capitalize on the opportunity to promote a feedback culture within the entire organization in which one improves through persistent hard work with input and mentoring from others [18,19].

### Tip 2: understand the limitations of SETs

Faculty and institutions should be familiar with the limitations and uses of SETs. Limitations include threats to validity, sources of bias, conflation with learner satisfaction, and other factors related to learner context and challenges [1, 20–22; 23]. Particularly for faculty with less contact with learners, poor learner recollection may undermine the accuracy of comments. In fact, in one study, students completed required evaluations including narrative comments for a fictional faculty [24]. Attention to biases related to gender, under-represented backgrounds, and part-time status are particularly important when SETs are used as a factor in advancement and promotion [1,20,22,25]. An observation that part-time teachers receive more critical comments than full-time teachers may warrant further investigation as a reflection of learner evaluation fatigue or a need to consider a threshold number of teaching hours to warrant productive evaluation. While SETs do have the potential to reflect learner outcomes [1,21], faculty and institutions should not interpret learner satisfaction as the sole marker of a faculty’s pedagogical competence and should be aware of the impact of other variables on satisfaction, such as factors related to innovation (Tip 3), learning context (Tip 6), and even faculty leniency. There is literature to suggest that faculty who challenge their learners or provide needed constructive feedback may receive poorer evaluations [26]. Conversely, faculty perceived by learners as being outstanding educators do not necessarily have better learning outcomes [27].

### Tip 3: recognize and account for the challenges of innovation

Curricular innovations deserve special consideration in the design and review of SETs as they are likely to generate critical feedback in the context of change. Implementing new content or practices involves a series of processes that allows the assimilation of an educational activity into an organization [28] and is a critical aspect in the continuous improvement of curriculum necessary for any educational program. However, the introduction of an innovation may create growing pains and implementation stutters that may lead to learner frustration with resulting harsh evaluations. The faculty and institution may overestimate learner readiness for new materials and tasks during the implementation phase of new curricula or, alternatively, the innovation may miss the mark.

While faculty generally recognized and embraced the need for implementation related to COVID and anti-racism, these innovations stretched them to work rapidly in areas outside of prior expertise [9]. In that setting, critical learner evaluations may be related to implementation challenges, and special consideration needs to be given to develop a thoughtful and rigorous evaluation process as well as systems of accountability [29]. Particularly during implementation, learner feedback provides an important, unique, and critical perspective on aspects of the learning environment and hidden curriculum that enable iterative improvement and ultimate success of the innovation [30].

Faculty and institutions can take several steps to help ameliorate the impact of implementation challenges on learners and improve the evaluation process: 1) provide clear instructions and set appropriate expectations for new content and methods, 2) explain the reason for the change and why it will help learning, 3) involve learners in the design and planning of the activity to foster learner agency, 4) create opportunities for early feedback that encourages students to separate their evaluation of the faculty from their evaluation of the innovation and 5) allow enough time for learners to adjust. In addition to these actions, institutions should anticipate the potential for critical feedback from learners during and following innovation and adjust expectations to allow this feedback to be a valued part of early phases of adoption. Failure to embrace critical feedback that is constructive during implementation could otherwise limit positive educational reform or lead institutions to abandon initiatives that would become valuable in time.

## Tips for faculty

### Tip 4: observe and reflect on one’s immediate response to feedback

Faculty should understand that it is common to experience strong emotional reactions to critical feedback in SETs, including shame, guilt, and anger [16,31,32] and develop a mindfulness-based approach to examining these reactions. A mindful approach to feedback includes openness and curiosity about the reaction, perspective-taking and letting go of judgment [17,33] and can reduce faculty fragility, in which marked discomfort and defensiveness impedes appropriate and productive actions and may lead to disengagement [34,35]. Since most SETs are in written form, faculty can first prepare by controlling the time and place in which they review them. Additional preparation involves assessing whether one is experiencing other feelings that might interfere with the ability to effectively receive the feedback, including being hungry, angry, lonely, or tired [36] When faculty take note of an emotional response, they may reflect on the trigger – was it a truth trigger, a relationship trigger, or an identity trigger [37]. A truth trigger could be characterized as a piece of feedback that feels discountable because it is untrue or inaccurate. Relationship triggers lead to discounting or defensiveness because of the perceived intent or characteristics (e.g., trustworthiness or credibility) of the feedback giver. Identity triggers are threatening or discordant with how one sees oneself. In reflecting on feedback, awareness of these triggers enables faculty to see past them to the potential opportunity for growth. As has been described for learner responses to feedback, managing affect and maintaining emotional equilibrium in the face of critical feedback enables a mindset that supports striving for continuous improvement [38].

### Tip 5: don’t overlook the positive

In analyzing data, faculty should be aware of the significance they give to individual pieces of feedback and the impact this may have on their development actions [4]. Some faculty have a tendency to attribute disproportionate weight or to fixate on critical comments [16]. Faculty may exhibit a sense of impostership and give greater significance to critical comments while discounting positive ones. Feelings of impostership can lock faculty into a static state, where one is less able to take chances, draw meaning from experiences or continue to improve [39]. In addition, faculty may introduce unjustified changes into their teaching in this case merely to please students [40].

Focusing on the negative not only results in a skewed perspective on faculty’s teaching but represents a missed opportunity to reflect and build upon strengths. Reinforcing feedback serves to illuminate behaviors that are working well, raising faculty awareness of effective methods to allow deliberate continuation of these behaviors. Reflecting on reinforcing feedback can also help faculty maintain a positive outlook and a continued growth mindset that enables more productive responses when constructive points arise and may even illuminate a strategy to address the area of necessary growth [17]. Finally, the presence of reinforcing feedback in complete contradiction to critical constructive feedback may point to an area that needs further exploration to understand varied student experiences and guide effective change [4].

### Tip 6: examine the learning context

Faculty should be aware that aspects of the learning context, including curricular timing and sequencing, environment (for example, clinical or classroom-based), and even broader sociopolitical climate may influence learning experiences and SETs. When critical evaluations cite instructional misalignment or problematic timing, faculty should be aware of the relation of the content to the broader curriculum. Has there been a change in a prior curricular component that had rendered the session more challenging (e.g., movement of a session that previously functioned as an introduction to the topic)? When learners question the relevance of a topic, consideration should also be given to the timing and location of the evaluation and whether learners are more likely to value the topic at a later stage [41] or different setting (e.g., evaluation of health systems science curriculum after starting clinical rotations). In other instances, the topic itself may be a factor. Is it innately challenging or polarizing? Is the challenging nature of the topic experienced disproportionately by a subset of students. For example, as mentioned in Tip 3, in the case of anti-racist curricular innovations, content will likely be experienced differently by persons of color than persons who are white [29]. In that case, discovering, exploring, and understanding the lack of equity in the learning experience will be critical to guide effective interventions and improvements.

### Tip 7: triangulate additional feedback data to make sound judgements about your work

Before taking action towards improvement, faculty should gather and triangulate evaluations of teaching with additional feedback data to make sound judgements about their work. Given the limitations of SETs, faculty benefit from additional data about the quality of their work. These data sources may include learning outcomes from assessments, faculty peer observation or coaching [42,43], or informal discussion with a colleague or mentor who can provide an additional perspective. The latter is not dependent on the availability of formal observation or coaching programs and can encourage socialization among teachers. As teaching is ultimately a solitary action, connections with colleagues have myriad potential benefits for educators by enabling the formation of communities of practice, defined as groups of people who interact on an ongoing basis to share concerns and engage in deepening their knowledge and expertise on common practices [44,45]. Finally, faculty should include in the available data their own self-assessment and personal development goals. Does the feedback relate to known areas of personal growth, or areas within the course that are in need of further development? Engaging local experts or those working in similar content areas for consultation and to learn from their experience may be helpful.

### Tip 8: take action with a focus on learning and learner agency

After taking time to reflect, contextualize feedback, and gather available data to make sound judgements, faculty should close the loop on the feedback with a focus on learning and learner agency. When the next steps are clear, communicating the actions being taken may be sufficient. When improvement steps are not immediately clear, the invitation for ongoing conversation is critical, and SETs can serve as a reminder that the educational effort benefits from productive communication. Conversations should focus learners on their learning, shifting them away from thinking about ‘likes’ and ‘dislikes’, and steer educators away from blaming learners for their dissatisfaction. Moreover, a focus on learning fosters a partnership with learners to improve the educational activity and better promotes achievement of key knowledge and skills. Moreover, partnership enhances learner agency, defined as their capacity to act purposefully to foster change [46]. By building learner agency, faculty contribute to the learners’ identities as changemakers, cultivate engagement in the learning endeavor, build self-efficacy skills, and enhance learning [47]. Increasingly, over the past decade, learner-led collaboration has become a core mechanism for curricular revision and innovation with some institutions formalizing a role for learner representatives in the continuous course review and improvement process [48].

## Tips for institutions

### Tip 9: provide active oversight of the evaluation process

Institutions or programs should provide active oversight of SETs that fosters alignment with the educational mission and includes mechanisms to learn from critical feedback. The standardized use of a set of core questions can facilitate consistency in data collection and allow for comparative data across courses, rotations, or curricular experiences. Core questions should reflect the value the institution places on certain teaching behaviors and learning outcomes. Attention to the wording of these questions can help focus learner responses on these behaviors and outcomes rather than on general satisfaction or a teacher’s personal characteristics, which are more susceptible to bias [27]. For instance, learners could rate the consistency between stated objectives and the content taught by a lecturer, a lab instructor’s organization of lab content, or a small group facilitator’s ability to facilitate group discussion and provide feedback.

Consideration should be given to the use of anonymous, confidential, and signed evaluations as well as the timing of collection. Anonymous evaluations facilitate honest upward feedback [49] for learners who might fear reprisal but may also enable comments that can feel hurtful [50,51]. Non-anonymous feedback, however, can lead to inflated scores and the loss of critical, constructive feedback [52,50,53,54]. A mechanism to provide feedback during the course can enable just-in-time adjustments when appropriate. Chosen approaches should be accompanied by learner and faculty development that fosters a culture of openness to feedback.

Finally, institutions and programs should actively monitor and review learner comments and have a process for identifying and responding to critical comments to support both faculty and students. If individual critical comments are unheeded in favor of the ‘majority’ voice, the institution risks eliminating the valuable feedback from learners in minoritized or historically oppressed groups. Institutional processes for responding should include consideration of how critical comments are communicated to the faculty to provide a supportive environment for reflection (Tip 4). Ultimately, feedback from SETs should help a faculty member grow as a teacher.

### Tip 10: develop learners’ ability to provide effective feedback

Institutions should develop learners’ abilities to complete evaluations in a manner that provides effective feedback to teachers. Learners may not understand the purpose of SETs or their role in continuous quality improvement. They may not consider the impact their comments can have when read by the individual under evaluation (versus a third party) and often do not realize that their evaluations have consequences for faculty promotion. Just as learners are expected to demonstrate competency in practice-based learning and improvement, which necessitates feedback literacy in their feedback response, they should similarly demonstrate feedback literacy in feedback provision. Learners should receive instructions on how to provide specific, skills-based feedback that helps faculty grow, that is professional and honest [11]. Creating a culture of bidirectional feedback [35] helps reduce hierarchy while providing faculty who are receptive to feedback an opportunity to learn and grow [55,56,57].

Institutions and programs may take a variety of approaches to promote feedback literacy. At minimum, they can provide basic guidelines for constructive and professional comments and train learners on effective ways to express their concerns to result in change. Some institutions specifically train a core group of learners to be the designated evaluators for their cohort, ensuring valid interpretations of the rating tool, rater reliability, and constructive comments [48,58,33]. Other institutions engage a learner leadership group in peer review of comments to help improve the quality and professionalism of student comments. Still other institutions have moved away from anonymous or confidential evaluations to signed evaluations to emphasize to learners that they ought not to put in writing anything they would be unwilling to say directly to a faculty member.

### Tip 11: develop and support faculty to respond constructively to feedback and provide faculty with resources to help them improve

Institutions should implement robust faculty development programs that foster constructive responses to feedback (see Tips 4–8; Esterhazy), improve teaching skills (i.e., the subject of the feedback), and create a growth mindset for continuous development. Care must be taken to not create a punitive culture around critical feedback. Faculty should feel supported to acknowledge difficulties or need for help or additional skills and feel capable of effecting change. Institutions must be sensitive to the challenges of developing faculty to teach in what may be perceived as drastically different ways (e.g., virtual strategies; anti-racist approaches to content and content delivery) while maintaining the accountability demanded of the work. By supporting faculty in their own learning actions (through connections to internal or external resources, such as those within a professional society), the institution can support a reflective practice amongst its educators that fosters improvement [59]. At the same time, institutions should recognize that some faculty may be demoralized by critical evaluations and feel their identity as an educator threatened. On rare occasions, both the institution and a faculty may realize that the faculty needs to step out of their teaching role. Institutions should have compassionate off-ramps for these circumstances when teaching is no longer a good fit.

### Tip 12: provide faculty with other evidence of teaching outcomes and implement a holistic system for evaluation of teaching

Whereas faculty can gather and triangulate data regarding their teaching (Tip 7), institutions can support faculty by putting processes in place to provide faculty with additional evidence of teaching quality beyond learner satisfaction. Decreasing the weight placed upon learner satisfaction ratings can encourage a more holistic evaluation of a faculty’s teaching contributions. For instance, institutions have data on learning outcomes from local and often national assessments with benchmarking data, which could be provided to faculty. Institutions can support peer observation programs for faculty to facilitate their ability to collect additional feedback and improve. By allowing the submission of an educator’s portfolio for promotion, institutions can enable faculty to communicate their educational philosophy and make their teaching visible [60]. The educator’s portfolio also allows faculty to provide context for their evaluations by including reflections on their intentional development plan. Expanding teaching data beyond learner evaluations and enabling faculty to articulate their teaching narrative helps ensure a more comprehensive picture of a faculty member’s teaching ability and quality while fostering their overall career development.

## Discussion

Learner evaluations of teaching can inform improvements when combined with steps that enable a productive response to critical comments. While studies have shown that SETs on the whole contain more positive reinforcing than critical comments, critical comments may engender negative emotional responses from faculty that have the potential to hinder the teaching development plan. In this time of rapid change in medical education due to the COVID-19 pandemic and amplified calls for anti-racism, creating a shared understanding of the role of feedback to guide improvement is all the more important. Students, faculty, and institutional leadership should understand the benefits and limitations of SETs, the potential for learning challenges during implementation processes, and the necessary steps to enable productive responses to learner feedback. Faculty should also be supported to develop skills for reflecting on their emotional response to feedback that hurts, approaches to triangulate sources of data on their teaching, and steps to enable a timely and appropriate response. Finally, institutions should provide oversight of the evaluation process, develop learners and faculty to effectively engage in feedback and provide faculty with evidence of their teaching outcomes that enable them to share their educational portfolio for advancement. This guide provides a framework for faculty and institutions to manage counterproductive effects of learner evaluation and productive steps to support key actions even in the face of words that hurt.
